# Novel deep intronic mutation in the coagulation factor XIII a chain gene leading to unexpected RNA splicing in a patient with factor XIII deficiency

**DOI:** 10.1186/s12881-019-0944-2

**Published:** 2020-01-08

**Authors:** Jun Deng, Dan Li, Heng Mei, Liang Tang, Hua-fang Wang, Yu Hu

**Affiliations:** 1 Institute of Hematology, Union Hospital, Tongji Medical College, Huazhong University of Science and Technology, Wuhan, Hubei People’s Republic of China; 2 Hubei Clinical and Research Center of Thrombosis and Hemostasis, Wuhan, Hubei People’s Republic of China

**Keywords:** Factor XIII, Deep intronic mutation, Coagulation disorders, RNA splicing, Nonsense mediated mRNA decay

## Abstract

**Background:**

Coagulation factor XIII (FXIII) plays an essential role in maintaining hemostasis by crosslinking fibrin. Deficiency in FXIII affects clot stability and increases the risk of severe bleeding. Congenital FXIII deficiency is a rare disease. Recently, we identified a Chinese family with FXIII deficiency and investigated the pathogenesis of congenital FXIII deficiency, contributing non-coding pathogenic variants.

**Methods:**

We performed common tests, coding sequencing by targeted next-generation sequencing (NGS), whole-genome sequencing and splice-sites prediction algorithms. The pathogenesis was investigated via minigene and nonsense-mediated mRNA decay (NMD) by experiments in vitro.

**Results:**

The proband is homozygote for a novel deep intronic c.799-12G > A mutation in the *F13A1* gene. Through direct sequencing of the minigenes mRNA, we found 10 bases of intron 6 insert in the mRNA of mutant minigenes mRNA. The relative expression of EGFP-F13A1 was higher by suppression of NMD in vitro. Furthermore, we found the proband with enhanced thrombin generation (TG).

**Conclusion:**

We reported a novel deep intronic c.799-12G > A mutation of *F13A1* which produced a new acceptor site and frame shifting during translation introducing a premature termination codon. Our results support the premature termination codon triggered NMD. We need to pay attention to the position of potential alterable splicing sites while counselling and genetic test. The finding of enhanced TG indicated that we should be aware of the risk of thrombosis in patients with FXIII deficiency during replacement therapy.

## Introduction

Congenital factor XIII deficiency is a rare disease with a prevalence of approximately 1 in 1–3 million individuals [1]. A higher incidence of the disease was observed in areas with frequent consanguineous marriages. Blood coagulation factor XIII (FXIII) was first recognized for its function. That is, it stabilizes fibrin clots in the concentrated urea solution [2, 3]. Therefore, FXIII was previously called fibrin-stabilizing factor, and it works in the final step of the coagulation cascade. The first is the rapid linear gamma fibrin chain crosslinking, followed by the slower crosslinking of the reticulated alpha fibrin chain, making the fibrin clot denser and firmer. FXIII consists of two A subunits and two B subunits that form tetramer circulation in the plasma. The A subunit has the main function, and it includes the catalytic core domain, activation peptide, calcium ion binding site, and other structure domains. The B subunit mainly acts as a carrier protein to stabilize the A subunit, connects the A subunit to fibrinogen, and down regulates the activity of FXIII. Apart from the hemostasis function of FXIII, it also maintains pregnancy, angiogenesis, bone biology, adipogenesis, and immunity [4].

Considering the function of FXIII, FXIII deficiency is a disease that poses a high risk for severe hemorrhage. Bleeding usually includes umbilical bleeding, prolonged bleeding after an injury or surgical procedure, subcutaneous bleeding, gum bleeding, intracranial bleeding, joint bleeding, and muscle bleeding [5, 6]. Statistics have indicated that intracranial hemorrhage is the most common cause of death [5]. Moreover, there are two kinds of FXIII deficiency, namely, congenital and acquired FXIII deficiencies. Congenital FXIII deficiency is a rare autosomal recessive bleeding disorder, and the unknown inhibitors in the plasma contribute to the acquired FXIII deficiencies as a complication of other diseases. Clinical history and discovery of genetic defects can help in easily distinguishing congenital and acquired FXIII deficiency. For FXIII deficiency, the results of conventional clinical tests, such as the test for platelet count, prothrombin time (PT), and activated partial thromboplastin time (APTT), are normal. Clot solubility assay is a qualitative test that helps identify if an individual has limited FXIII activity in the plasma. However, a missed diagnosis may occur when in patients with mild or moderate FXIII deficiency [6]. We usually compare the FXIII activity or FXIII antigen level of individuals with such deficiency to that of normal individuals. When the FXIII activity or FXIII antigen level is low, corresponding genes sequencing can help identify the variation. In cases of congenital FXIII deficiency, mutations in the A and B subunits account for 95 and 5% of all mutations, respectively [7].

Among the mutations of the A subunit reported in the HGMD database, most are missense or nonsense mutations, and only 10% are splicing site mutations. During the process of splicing, specific sites in the precursor mRNA are required, which include a donor site, branch site, and acceptor site. The altering of splicing site can affect mature mRNA. This may produce abnormal protein and affect normal function. To protect cells from potential harm by aberrant mRNA, nonsense-mediated mRNA decay (NMD) plays a significant role by degrading mRNA that contain premature stop codons.

Recently, a patient with history of severe bleeding was diagnosed with congenital FXIII deficiency. To identify the underlying molecular mechanism, we assessed her family members and found a novel deep intronic mutation of *F13A1* through next-generation sequencing (NGS). Meanwhile, we found nearly no corresponding mRNA expression through RNA sequencing (RNA-Seq). To explore the reason, we utilized prediction algorithms to predict the potential function of this mutation. Using minigene and interference in NMD, we showed the generation of a new splicing site that produced premature termination codon that induced NMD. In addition, an interesting finding showed that patients with low FXIII levels presented with enhanced thrombin generation.

## Methods

### Patient and her family

One female patient with an inherited bleeding syndrome was included in the study. We evaluated the severity of bleeding based on the ISTH-BAT score [8]. Blood coagulation test was conducted, and the activity of coagulation factors and anticoagulation proteins was assessed. Urea clot lysis test and factor XIII antigen (FXIII Ag) test with an automated latex enhanced immunoassay were carried out for the quantitative determination of factor XIII Ag in the citrated plasma using the IL Coagulation Systems (STA-R Evolution, Stago). Pedigrees analysis was conducted for her family.

Informed consent for medical diagnosis and research was obtained from the patient and her relatives. This study was approved by the ethics committee of the institutional review board at Union Hospital, Tongji Medical College, Huazhong University of Science and Technology.

### Thrombin generation assay

Blood samples were collected and placed in the BD Vacutainer® blood specimen collection tube containing 0.106-M sodium citrate (final dilution 1:10). Platelet-poor plasma was prepared via centrifugation at 2200 g for 15 min at room temperature. The samples were stored at − 80 °C until tested.

Thrombin generation was measured using the calibrated automated thrombinography (CAT) method (Thermolab Systems, Finland), with a tissue factor of 1 pM (FLUCA KIT; Stago). The detailed protocols have been described, as shown by Hemker et al. [9]. The thrombin generation assay (TGA) parameters, such as lag time (LT), peak thrombin (peak), endogenous thrombin potential (ETP), and start tail time, were generated using the CAT software (Thrombinoscope BV, Maastricht, The Netherlands).

### DNA mutation detection

Genomic DNA was isolated from peripheral blood leukocytes using the QIAamp DNA mini kit (Qiagen, Hilden, Germany). We used targeted NGS with a self-designed panel, not only to detect the mutation in *F13A1* and *F13B* gene for the proband but to exclude other common inherited bleeding disorders. This panel was designed to capture all the protein-coding regions and 10 bp of flanking intronic sequence of 70 genes, which involved most of inherited bleeding, as well as thrombotic and platelet disorders. We also performed whole-genome sequencing of the three family members with the BGISEQ-500 sequencing system (Beijing Genomic Institution, Shenzhen, China). PCR and Sanger sequencing were performed with the leukocyte DNA of the patient’s family members to validate genetic variation found via NGS. We referred to HGMD-Professional-release- 2019.3 database and ClinVar_20191101 database to determine whether the mutation was novel or not.

### Splice-site predictions

To identify the potential impact of the c.799-12G > A variant on splicing, the Human Splicing Finder was used [10].

### Construction of the F13A1 minigene vector

The construction and validation of the minigene used in this study to verify *F13A1* c.799-12G > A mutation resulting in unexpected RNA splicing has been widely accepted [11]. Briefly, a 442-bp PCR fragment containing exon 7 and its adjacent intron 6 were amplified from wide-type and mutated human genomic DNA. The 12 sequences used were as follows: 5′ -ggtaggtacccacactcctcctatctg-3′ and 5′ -tgcagaattcatgtgttaaagacacca-3′ (restriction sites underlined). After restriction of enzyme digestion of used plasmid by KpnI and EcoRI, PCR products were inserted into the pcMINI plasmid. Finally, all minigene constructs were sequenced to verify the correct insertion of the wild-type and mutated DNA fragments.

### Cell culture and transfection

Hela and 293 T cells were cultured in DMEM (Gbico,USA) with 10% fetal bovine serum at 37 °C in 5% CO_2_. Plasmids were transfected using the Liposomal Transfection Reagent (Yeasen, Shanghai, China).

### RT-PCR and mRNA analysis

Total RNA was extracted from 293 T or Hela cells 48 h after transfection and was reverse transcribed to cDNA using the GoScript™ Reverse Transcription System kit (Promega, Wisconsin, the USA). The 5′ and 3′ flanking regions (279 bp) of the splicing mutation site (c.799-12G > A) were amplified via PCR using GoTaq Master Mixes (Promega) and the following protocol: initial denaturation at 94 °C for 5 min, followed by 40 cycles at 94 °C for 15 s, 60 °C for 25 s, and 72 °C for 1 min, with a final elongation at 72 °C for 5 min. The PCR products were electrophoresed on a 1.8% agarose gel and were sequenced. The primers were as follows: 5′ -gggaggtggatgttcaaggcagca-3′ and 5′ -tgcagaattcatgtgttaaagacacca-3′.

### Construction of the F13A1-c.798 + 1ins expression vector and transfection

The F13A1 coding sequence was amplified via PCR from a F13A1 cDNA plasmid (Sino Biological, Beijing, China). Using overlap extension PCR, 10 base pair was introduced into the end of exon 6 to obtain the fragment of F13A1-c.799-12G > A. This product was inserted into the pGFP expressing vector. All constructs were sequenced to verify the correct insertion of the wild-type and mutated DNA fragments.

Human embryonic kidney (293 T) cells were transfected with a mutant (mut) or a wild-type (wt) pGFP-F13A1 vector, as described above. After 48 h, the expression of GFP-F13A1 protein was detected using a fluorescence microscope. Total RNA and proteins were extracted and analyzed via qPCR and western blot. Transfected 293 T cells were also treated with 20 μg/mL of cycloheximide (CHX), a translation inhibitor, for 8 h. Total RNA was analyzed via qPCR.

### RNA interference

To inhibit NMD through the UPF1 pathway, we purchased an UPF1 small interfering RNA (UPF1 siRNA) from Ribobio (Guangzhou, China). Moreover, 100 nM siRNA and 5 μg plasmids were transfected into 293 T cells for 48 h. Total RNA and proteins extracts were obtained from two 10-cm plates of 293 T cells per condition according to the manufacturers’ instructions.

### Real-time PCR

The total RNA was reverse transcribed to cDNA, as described above. QPCR was performed on ABI 7500fast system using the SYBR Green Realtime PCR Master Mix kit (Thermo, Massachusetts, the USA). The relative abundance of target mRNA was normalized to GAPDH, and mRNA levels in cells expressing the pGFP-F13A1-wt were set to 100%. The following primers were used for qPCR: 5′-acacccactcctccaccttg-3′ and 5′-ctcttcctcttgttgctcttgctg-3′ for GAPDH; 5′-atcatggcctacaagcagaa-3′, and 5′-tctcgttggggtctttgct-3′ for GFP; 5′-agaggtgaccctgcacaagg-3′, and 5′-agccgaggaggaagacgttg-3′ for UPF1.

### Western blot

In total, 10 μg of total proteins were separated on a 12% SDS-PAGE and transferred to a polyvinylidene-fluoride membrane. The proteins were probed with a monoclonal mouse anti-UPF1 and anti-GFP (Cell Signaling Technology, Massachusetts, the USA). The membranes were stripped and re-probed with an anti-β actin as a control.

### Statistical analysis

All values were presented as mean ± SD. Differences between the mutation group and the wild group were compared by the unpaired t test or Welch’s t-test when appropriate. *P* value < 0.05 was considered statistically significant in all analyses.

## Results

### Characteristic features of F13-deficient patients and her family

**Fig. 1:**
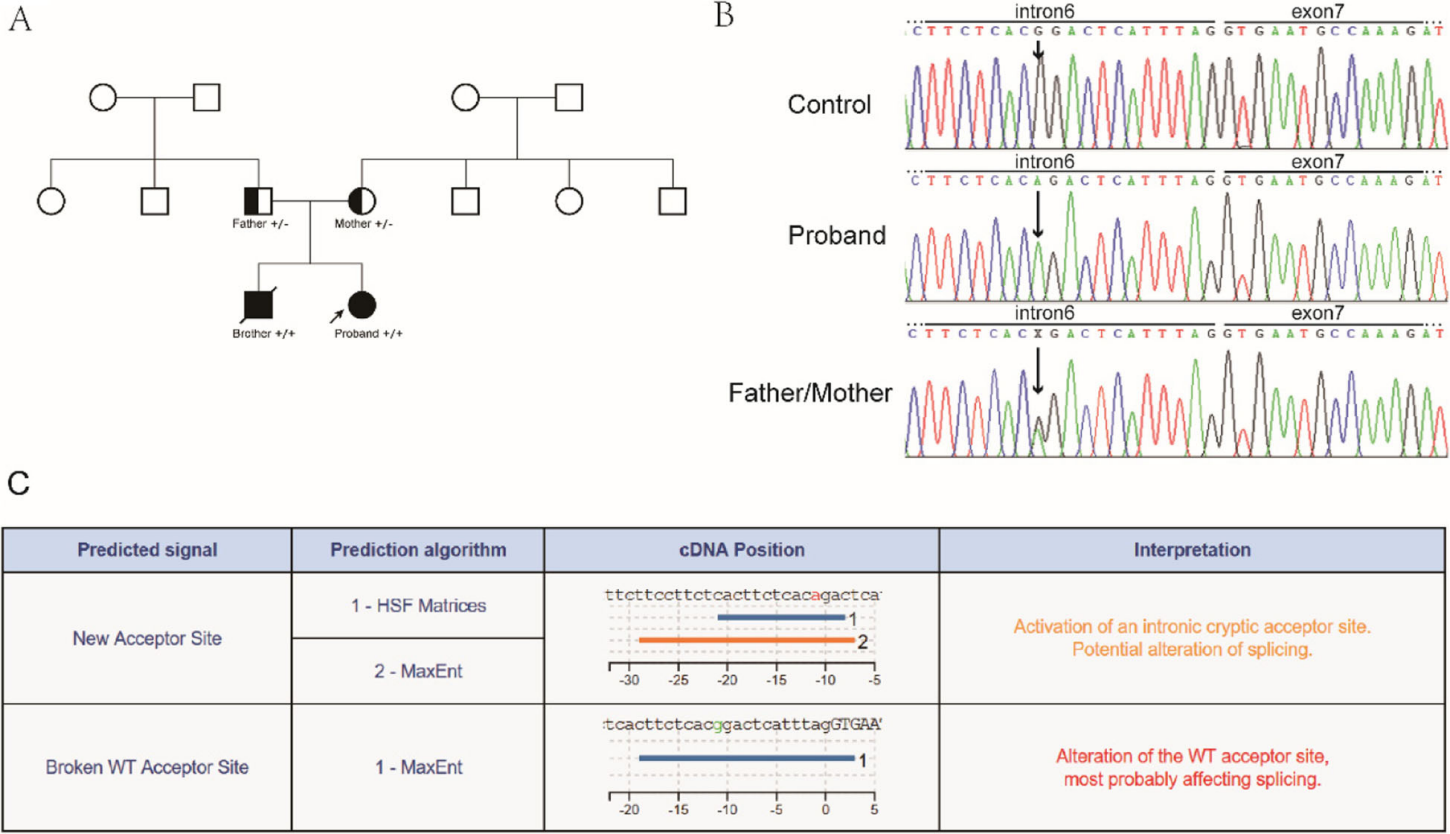
Identification of *F13A1* mutation. **A**, the family tree. **B**, Sanger sequences for the proband (homozygote), mother/father (heterozygote), and unaffected control. **C**, the results of Human Splicing Finder. Both prediction algorithms of the new acceptor site indicate the potential alteration of splicing

There are two affected patients in this family (pedigree in Fig. 1a). The parents denied consanguineous, and the brother died of intracranial hemorrhage at 2 years of age. The proband, who is now a young adult, underwent initial F13 investigations at the age of 5 years. She was diagnosed with FXIII deficiency, and we also confirmed this in our center via antigen of FXIII and urea test. Musculoskeletal hematomas and poor wound healing were observed. She had normal PT and APTT but extremely low level of FXIII Ag. Moreover, the proband had abnormal urea test result (Table 1). She had not received routine replacement therapy. However, she received transfusion of fresh frozen plasma once because she underwent unilateral ovariectomy at the age of 14 years due to heavy bleeding caused by corpus luteum rupture. The ISTH-BAT-bleeding score for the proband was 10. No other associated syndromes were observed. Both her mother and father had a mild decrease in FXIII Ag levels and were asymptomatic. Furthermore, the proband and her family did not receive any medications before the tests were conducted.

**Table 1:** Characteristics and laboratory test results of the patient

Individual	Age	Mutation type	PT (s)	APTT (s)	FXIII Ag (%)	Urea test	Clinical information
Proband	27	Homozygote	12.1	30.7	< 2.4↓	Clot lysis within 2 h	Bleeding diathesis
Mother	53	Heterozygote	11.9	35.0	64.5↓	Negative	Asymptomatic
Father	55	Heterozygote	11.9	34.4	60.9↓	Negative	Asymptomatic

Reference ranges: PT, 10.0–16.9s; APTT, 20.0–43.5s; FXIIIAg (%), 75–

155%

Abbreviations: *APTT* activated partial thromboplastin time; *PT* prothrombin time; *FXIII* Ag, antigen of coagulation factor XIII

Note: ↓, reduced

### Unexpected generation of high thrombin levels in FXIII-deficient patients

**Fig. 2:**
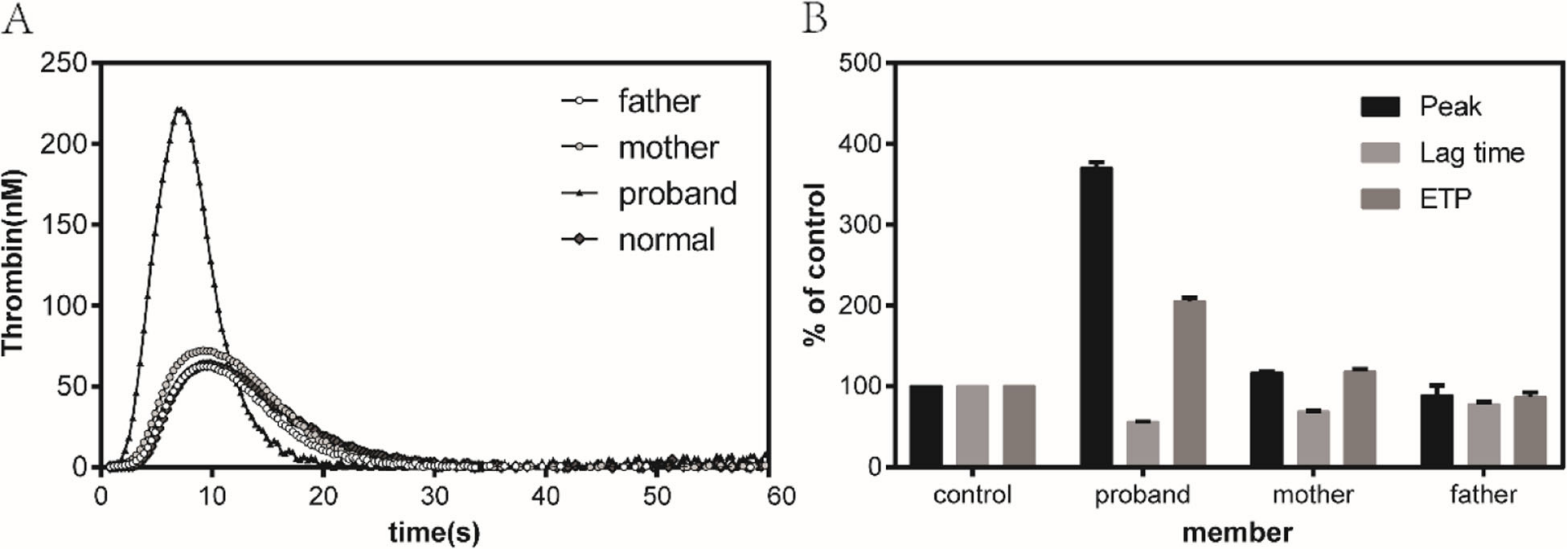
High level of thrombin generation for the proband. **A**, representative thrombin generation curves for the proband and her parents in the plasma spiked with 1 pM tissue factor. **B**, ETP (black bars), peak thrombin generated (light bars), and LT (dark gray bars) of the plasma of the trio family were shown as a percentage of normal control plasma

To investigate whether FXIII protein has an impact on thrombin generation, we performed TGA using the calibrated automated thrombography method. Interestingly, the thrombin generation of the proband was higher than that of her parents and normal control (Fig. 2a and b). Both her peak thrombin generation and ETP showed were two times higher than those of the normal controls. Her LT was not significantly altered. However, the ETP, peak thrombin generation, and LT of the parents did not significantly differ compared with normal controls.

### A novel splicing mutation detected in F13A1

With a self-designed panel, no mutation was observed in the coding sequence of *F13A1* and *F13B* gene. To determine the molecular alterations that caused FXIII deficiency, we performed whole-genome sequencing for the trio family. Based on congenital F13D with a recessive genetic pattern, we first identified all mutations in F13A and F13B genes of the proband and her parents, then filtered out mutation sites with a frequency of more than 1%, and finally identified a novel homozygous c.799-12G > A mutation in intron 6 of *F13A1* in the proband, whereas no mutations were found in the *F13B* genes. Next, we confirmed the mutation in the patient and her family members via direct sequencing (Fig. 1b). Both her parents are heterozygous for the same mutation in *F13A1*. The effect of the predicted splicing variant (c.799-12G > A) was first predicted using the Human Splicing Finder tool. The prediction algorithms showed that c.799-12G > A may be a variant that most probably affected splicing and activated an intronic cryptic acceptor site (Fig. 1c).

### Altered splicing study of F13A1 in the proband

**Fig. 3:**
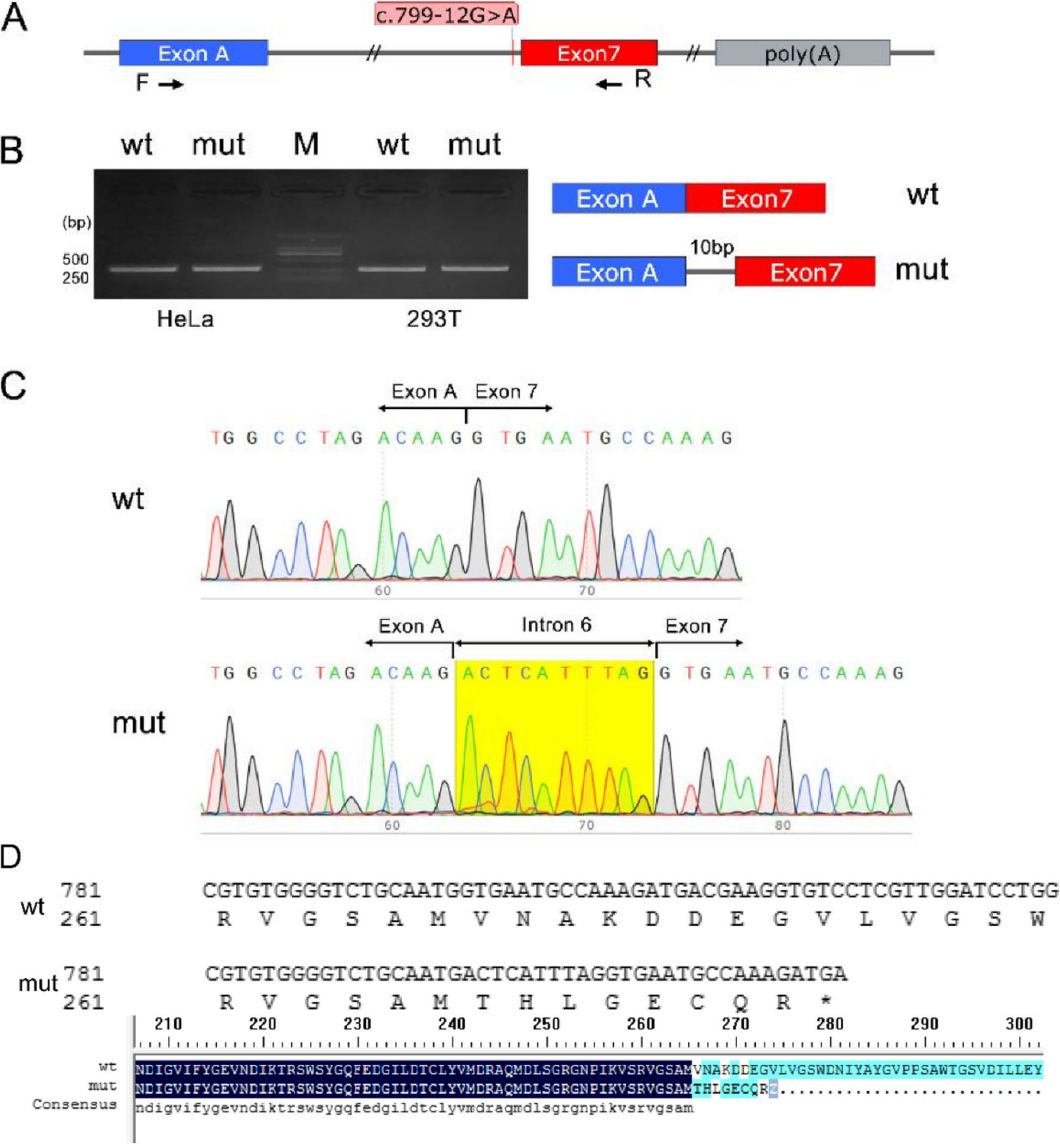
Analysis of the c.799–12 G > A mutation using minigene construct. **A**, the F13A1 gene fragment selected for minigene. The position of mutation site c.799-12G > A and fragment containing exon 7 and its adjacent intron 6 are indicated. **B**, **C** analysis of mRNA from transfected Hela and 293 T cells via real-time polymerase chain reaction and direct sequencing. The expressed WT and mutant minigenes mRNA was analyzed via agarose gel electrophoresis and direct sequencing. **D**, The amino acid sequence of wt and mutated protein

To verify the splicing effect of mutation c.799-12G > A in vitro, minigene assays were performed. Minigene-wt and minigene-mut represent the genotypes of wide-type and homozygous mutations in c.799-12G > A, respectively (Fig. 3a). Each minigene was transfected into the Hela and 293 T cells. The expressed wt mRNA and mut mRNA were analyzed via agarose gel electrophoresis (Fig. 3b) and direct sequencing (Fig. 3c). The last 10 nucleotides of intron 6 appeared in the mutant mRNA.

### In vitro gene expression

**Fig. 4:**
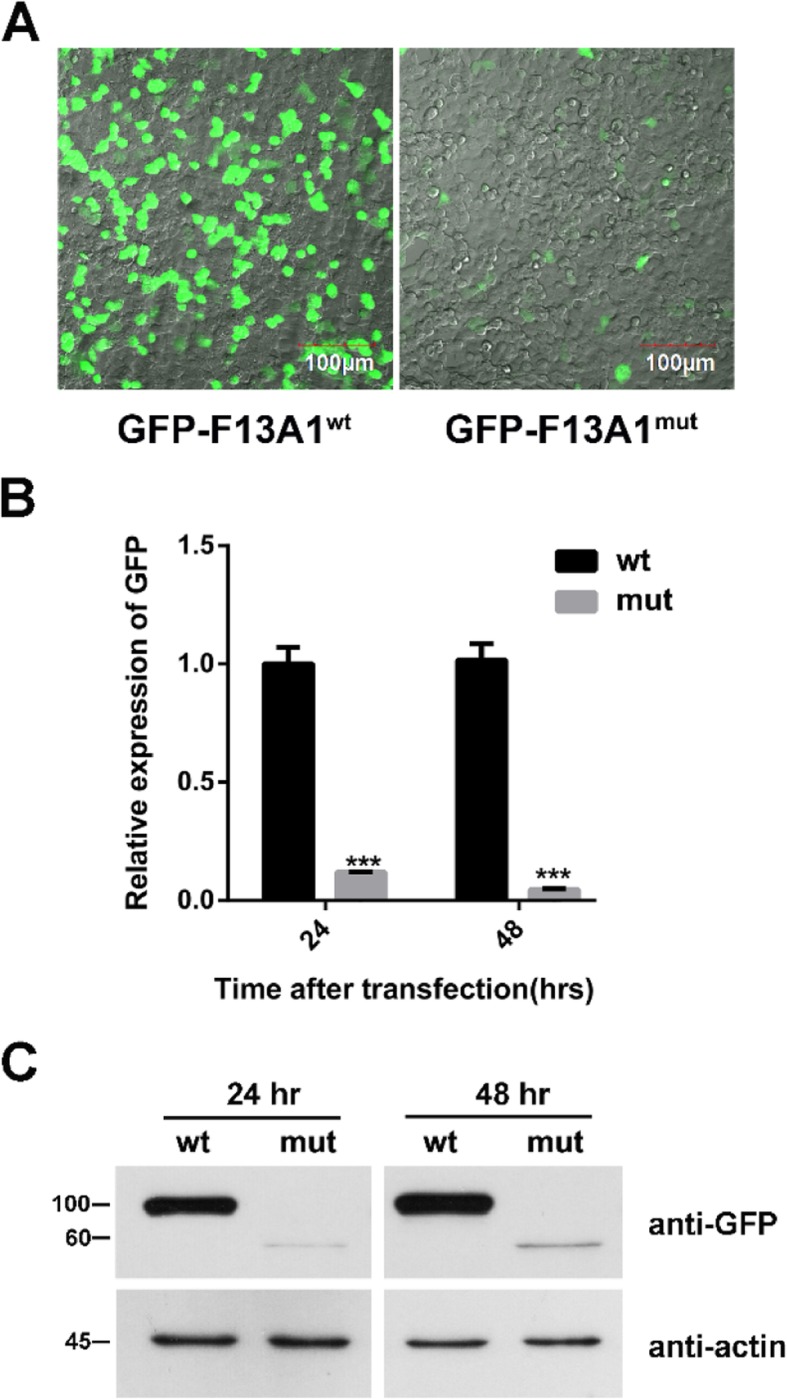
GFP-F13A1 expression in transfected cells. **A**, 293 T cells were transfected with wt and mut plasmids for 48 h. Fluorescent signals of GFP-F13A1 were detected via fluorescent microscopy. 293 T cells were transfected with wt and mut plasmids. **B,C**, GFP-F13A1 mRNA and protein expression were quantified via qPCR and western blot ***p < 0.01.

To investigate the effects of the mutations c.799-12G > A on F13A1 expression, plasmids expressing mutant-type (mut) and wild-type (wt) EGFP-F13A1 cDNA was transfected into 293 T cells, respectively. After 48 h, the expression of EGFP-F13A1 protein was detected using fluorescent microscope. The fluorescence signals produced by EGFP-F13A1-mut were significantly weaker than those of EGFP-F12A1-wt (Fig. 4a). Consistent with detection using florescent microscope, the mRNA transcribed from pGFP-F13A1-mut was significantly lower than that from the pGFP-F13A1-wt, and the mRNA levels expressed by mut plasmids were 12 and 5% of the wt plasmids at 24 h and 48 h, respectively (Fig. 4b). Accordingly, the expression of mutant EGFP-F13A1 proteins was also lower than that of the wild-type EGFP-F13A1 protein (Fig. 4c). The molecular size of EGFP-F13A1-mut was smaller than that of wild-type protein, indicating that c.799-12G > A mutation can cause a truncated protein. The amino acid sequence of wt and mutated protein showed in Fig CAT software.

### Effects of the suppression of NMD by CHX

**Fig. 5:**
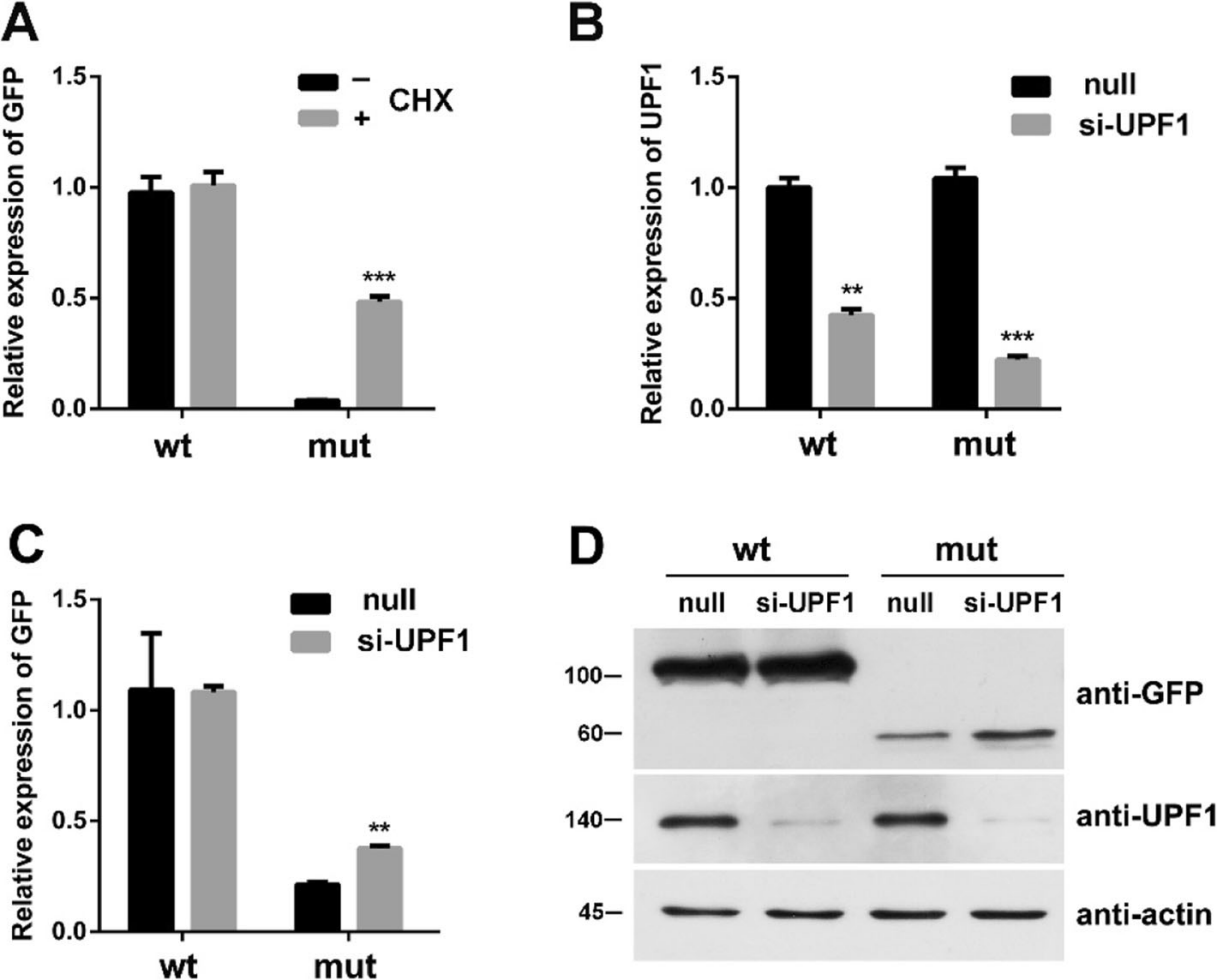
Effects of the suppression of NMD by CHX and UPF1 siRNA (si-UPF1). **A**, 293 T cells were transfected with WT and mutant plasmids and were sequentially added into CHX to inhibit translation for 8 h. The relative expression of EGFP-F13A1 was verified via qPCR. **B**, the expression of UPF1 was analyzed using transfected cells by si-UPF1. **C, D**, 293 T cells expressing the WT and mutant F13A1 were transfected with UPF1 siRNA for 48 h. EGFP-F13A1 mRNA and protein expression were quantified via qPCR and western blot. **p < 0.05, ***p < 0.01

To assess whether the decrease in the abundance of mRNA levels in the c.799-12G > A mutation is due to NMD, 293 T cells were transfected with WT and mutant plasmids and were sequentially added into CHX for 8 h. The relative expression of EGFP-F13A1 was verified via qPCR. CHX is an inhibitor for translation, and it can suppress the NMD pathway to degrade mutant mRNA [12]. The mRNA transcribed from pEGFP-F13A1-mut was significantly lower than that of the pEGFP-F13A1-wt (Fig. 5a). These results indicated that CHX inhibited NMD and protected the mutant EGFP-F13A1 mRNA from degradation.

### Effect of the suppression of UPF1 on the NMD of the c.799-12G > A mutation

The UPF1 protein was proven to be a key factor for NMD [13]. Knocking-down UPF1 expression by RNA interference (RNAi) has been used as a functional assay to assess the NMD sensitivity of mRNA transcripts that contain premature termination codons. To investigate the role of UPF1 in the degradation of mRNA of mutant F13A1, we used a siRNA to inhibit the expression of UPF1. In these experiments, 293 T cells expressing the WT and mutant F13A1 were transfected with siRNA. The UPF1 knockdown in the transfected cells was confirmed via qPCR and western blot (Fig. 5b and d). Detection of actin with anti-actin antibody served as a loading control. For the F13A1 mRNA transcripts, the level of c.799-12G > A mutant mRNA significantly increased in UPF1-siRNA transfected cells (Fig. 5c). These results indicated that mRNA transcripts of the c.799-12G > A mutation can induce NMD.

## Discussion

Congenital FXIII deficiency is a rare autosomal disease, which is more commonly diagnosed in areas with massive consanguinity. Individuals with severe FXIII deficiency present with severe bleeding with a high risk of life-threatening bleeding. In the present study, the clinical symptoms of the proband and her brother described above were in accordance with the characteristic symptoms of FXIII deficiency. Furthermore, the proband scored 10 points in the ISTH/SSC bleeding assessment tool [8]. In addition to the commonly reported symptoms, the proband experienced hemoperitoneum after a unilateral spontaneous corpus luteum rupture at the age of 14 years. She underwent unilateral oophorectomy to achieve hemostasis. Severe intraperitoneal hemorrhage secondary to ovulation or corpus luteum rupture is rare but can occur in individuals with congenital coagulation disorders [14]. Early diagnosis and treatment can effectively prevent unnecessary surgeries and preserve fertility in these individuals.

In this study, we primarily aimed to present a novel deep intronic mutation in the *F13A1* gene and to assess the pathogenic mechanism underlying such mutation, which was found to be relatively rare. At first, we did not find any mutation in the coding sequence until we performed whole-genome sequencing. Eventually, we identified a novel homozygous c.799-12G > A mutation in intron 6 of the *F13A1* gene in the proband. Then, we confirmed that both her parents were heterozygous for the same mutation. Among the *F13A1* gene defects, the most frequently reported mutations were missense in exons, whereas mutations in introns only accounted for approximately 11% of all mutations [15]. Using several prediction algorithms tools, we predicted that this mutation might generate a new splice site, which could be verified by minigene [16]. In vitro, we utilized a minigene strategy to simulate physiological process to extract the mRNA of the designed minigenes. We confirmed that a 10 bp of intron 6 was detained in the mutant mRNA due to the new splice site by direct sequencing (Fig. 3c). However, comparing transcriptome sequence results of the trio family, we detected the lack of mutation gene expression (data not shown). The detained 10 bp in the mRNA caused frame shifting, which may produce a premature termination codon-triggering NMD.

NMD is a protection mechanism that prevents potentially deleterious truncated protein production, which can be blocked down by CHX and knockdown of Upf1 by siRNA [17, 18]. Both kinds of translation inhibition result in the restoration of mutant mRNA to levels comparable to the WT minigene (Figs. 4 and 5). Our results indicated that NMD causes massive reduction of mutant mRNAs. Similar methods that verify the occurrence of NMD are commonly found in the literature [19, 20].

Moreover, we found an unexpected phenomenon in which the thrombin generation (TG) capacity of the proband is particularly strong. She did not receive any therapy before the tests. However, her thrombin peak height is more than twice the value for normal controls, and her ETP is also greater than that of normal controls. In another FXIII-deficient patient, the peak value was also double (data are not shown). About this enhanced TG, Hanna et al. have reported the phenomenon and findings indicating that low FXIII levels impair the structure and function of fibrin and simultaneously enhances TG to support hemostasis [21]. In addition to this, Almeida et al. [22] have reported about a 3-year-old girl with FXIII deficiency who presented with deep venous thrombosis. We assumed that the long-term lack of FXIII leads to an increase in the compensation of other coagulation factors and procoagulant components. Thus, more studies must be conducted to verify this assumption and to identify the relationship between low FXIII levels and enhanced TG. We will also assess such relationship.

## Conclusions

In conclusion, the patient with a homozygous mutation was diagnosed with congenital FXIII deficiency. The novel deep intronic c.799-12G > A mutation of *F13A1* produced a new acceptor site leading to 10 bp of intron 6 detained in the mutant mRNA and frame shifting during translation which introduced a premature termination codon. Our results support then notion that the premature termination codon triggered NMD. The deep intronic mutation shows that the mutations in the introns, particularly in the position of potential alterable splicing sites. Thus, we must pay attention to this. Some patients still presented with low FXIII levels and enhanced TG. This finding indicated that we must be aware of the risk of thrombosis in patients with FXIII deficiency during replacement therapy. We will continue to assess the relationship between low FXIII levels and enhanced TG.

## Data Availability

The datasets used and/or analysed during the current study are available from the corresponding author on reasonable request. Relevant DNA sequencing data can be found online at https://www.ncbi.nlm.nih.gov/bioproject/PRJNA596625.

## Abbreviations

APTT: Activated partial thromboplastin time
CAT: Calibrated automated thrombinography
CHX: Cycloheximide
ETP: Endogenous thrombin potential
FXIII Ag: Factor XIII antigen
FXIII: Factor XIII
LT: Lag time
NGS: Next-generation sequencing
NMD: Nonsense-mediated mRNA decay
PT: Prothrombin time
RNA-Seq: RNA sequencing
TG: Thrombin generation
